# Early introduction of selective immunosuppressive therapy associated with favorable clinical outcomes in patients with immune checkpoint inhibitor–induced colitis

**DOI:** 10.1186/s40425-019-0577-1

**Published:** 2019-04-02

**Authors:** Hamzah Abu-Sbeih, Faisal S. Ali, Xuemei Wang, Niharika Mallepally, Ellie Chen, Mehmet Altan, Robert S. Bresalier, Aline Charabaty, Ramona Dadu, Amir Jazaeri, Bret Lashner, Yinghong Wang

**Affiliations:** 1 Department of Gastroenterology, Hepatology and Nutrition, The University of Texas MD Anderson Cancer Center, Unit 1466, 1515 Holcombe Blvd, Houston, TX 77030 USA; 2 Department of Biostatistics, The University of Texas MD Anderson Cancer Center, Houston, TX USA; 3 Department of Medicine, Baylor College of Medicine, Houston, TX USA; 4 Department of Thoracic/Head and Neck Medical Oncology, The University of Texas MD Anderson Cancer Center, Houston, TX USA; 5 Department of Gastroenterology, Hepatology and Nutrition, MedStar-Georgetown University Hospital, Washington, DC USA; 6 Department of Endocrine Neoplasia and Hormonal Disorders, The University of Texas MD Anderson Cancer Center, Houston, TX USA; 7 Department of Gynecologic Oncology and Reproductive Medicine, The University of Texas MD Anderson Cancer Center, Houston, TX USA; 8 Department of Gastroenterology, Hepatology and Nutrition, Cleveland Clinic Foundation, Cleveland, OH USA

**Keywords:** Colitis, Diarrhea, Immune checkpoint inhibitors, Immunotherapy, Infliximab, Vedolizumab

## Abstract

**Background:**

Current treatment guidelines for immune-mediated colitis (IMC) recommend 4 to 6 weeks of steroids as first-line therapy, followed by selective immunosuppressive therapy (SIT) (infliximab or vedolizumab) in patients who do not respond to steroids. We assessed the effect of early SIT introduction and number of SIT infusions on clinical outcomes.

**Methods:**

We performed a retrospective review of patients with IMC who received SIT at The University of Texas MD Anderson Cancer Center between January and December 2018. Logistic regression analyses were used to assess associations between clinical outcomes and features of IMC.

**Results:**

Of the 1459 patients who received immune checkpoint inhibitors, 179 developed IMC of any grade; 84 of these 179 patients received SIT. Of the 84 patients who received SIT, 79% were males, and the mean age was 60 years (standard deviation, 14). Compared with patients who received SIT > 10 days after IMC onset, patients who received early SIT (≤10 days) required fewer hospitalizations (*P* = 0.03), experienced steroid taper failure less frequently (*P* = 0.03), had fewer steroid tapering attempts (*P* < 0.01), had a shorter course of steroid treatment (*P* = 0.09), and had a shorter duration of symptoms (*P* < 0.01). Patients who received one or two infusions of SIT achieved histologic remission less frequently (*P* = 0.09) and had higher fecal calprotectin levels after SIT (*P* = 0.01) compared with patients who received three or more infusions. Risk factors for IMC recurrence after weaning off steroids included: 1) needing multiple hospitalizations, 2) experiencing steroid taper failure after SIT, 3) receiving infliximab rather than vedolizumab, 4) receiving fewer than three infusions of SIT, 5) having higher fecal calprotectin levels after SIT, and 6) receiving a longer course of steroids, hospitalization and IMC symptoms. Unsuccessful weaning from steroids after SIT was associated with high IMC grades; multiple hospitalizations; steroid-resistant IMC; long interval from IMC to SIT initiation; and long duration of steroids, IMC symptoms, and hospitalization.

**Conclusion:**

SIT should be introduced early in the disease course of IMC instead of waiting until failure of steroid therapy or steroid taper. Patients who received three or more infusions of SIT had more favorable clinical outcomes.

**Electronic supplementary material:**

The online version of this article (10.1186/s40425-019-0577-1) contains supplementary material, which is available to authorized users.

## Introduction

The advent of immune checkpoint inhibitor (ICI) therapy revolutionized the landscape of advanced cancer therapeutics. As the use of ICIs increases and gains global access, the need for updated management recommendations for the immunotherapy-related adverse events (irAEs) associated with ICI therapy becomes pivotal to the safety profile of ICIs. Recently, the American Society of Clinical Oncology, National Comprehensive Cancer Network, European Society for Medical Oncology, and Society for Immunotherapy of Cancer established guidelines for the management of irAEs [1–4]. These recommendations, however, are based on a limited body of evidence. Treatment of irAEs is not well defined for every clinical setting, and some questions remain unanswered. Therefore, further investigation regarding the management of these irAEs is merited.

Immune-mediated colitis (IMC) is among the most commonly encountered irAEs that lead to ICI treatment discontinuation [5, 6]. IMC can be devastating and can adversely affect the quality of life of cancer patients. Similarities as well as divergences have been observed between IMC and idiopathic inflammatory bowel disease (IBD) in terms of pathogenesis, clinical presentation, and endoscopic and histopathological features [7–9]. Taking into consideration the mixed nature of the immune infiltrate found in IMC, rich in T cells and neutrophils [10], the use of selective immunosuppressive therapy (SIT) seems an appropriate frontline strategy for a dual purpose: (1) to treat IMC more quickly and (2) to avoid relapse during corticosteroid tapering. These observations form the rationale behind transposing treatment approaches for IMC from the IBD field. Thus, an anti-T cell blockade by vedolizumab or an anti-neutrophil blockade by infliximab through tumor necrosis factor are appropriate therapies for this purpose.

Current treatment guidelines recommend corticosteroid therapy as first-line therapy for IMC and that corticosteroids be continued for at least 4 to 6 weeks after resolution of IMC [1–4]. This approach could expose patients to unnecessary immunosuppression and increases the risk of corticosteroid-associated morbidity. The introduction of SIT, such as infliximab and vedolizumab, is currently recommended only after failure of corticosteroids to induce resolution of symptoms. No data are available on the appropriate timing of SIT administration and the effect of early introduction of SIT before steroid failure. Moreover, recommendations regarding the optimal number of SIT infusions is lacking. These knowledge gaps have not been addressed and are an obstacle in the path of evidence-based recommendations for the management of IMC.

Previously, we published data regarding the utility of endoscopic and histologic evaluation in the management of IMC and the importance of prompt evaluation to guide early SIT initiation [7, 11, 12]. We also found investigational laboratory tests to be useful in predicting the response to immunosuppressive therapy. Additionally, we reported the effectiveness and safety of vedolizumab and infliximab in the treatment of IMC [13, 14]. These studies of the endoscopic, histologic, and therapeutic paradigms of IMC were inspired by the experience with the treatment approaches for IBD and yielded findings that are akin to IBD. The current study builds upon this foundation.

At a tertiary cancer center, we are frequently consulted for the treatment of IMC that is refractory to corticosteroid therapy. Delay in SIT introduction and inadequate number of SIT infusions have been shown to be associated with significant morbidity of uncontrolled IBD and serious sequelae [15], and our clinical observation suggests a similar trend with IMC patients. Therefore, we sought to define the impact of early introduction of SIT, number of SIT infusions, and the duration of steroids on the clinical outcomes of IMC, aiming to contribute to the body of evidence for the upcoming IMC treatment guidelines.

## Methods and materials

### Patient population

Approval for this retrospective study was obtained from the institutional review board at The University of Texas MD Anderson Cancer Center. We included adult patients who developed IMC and received SIT in 2018. We excluded patients with colitis due to other etiologies, including infectious colitis, graft-versus-host disease, and neutropenic colitis. Institutional pharmacy, endoscopy, oncology, and gastroenterology databases were searched to identify eligible patients. Thereafter, a comprehensive chart review was conducted to extract variables of interest.

### IMC information

Data pertaining to IMC were date of onset, time from ICI initiation to onset, duration of symptoms, presenting and peak grades of diarrhea and colitis, treatment, and outcomes. IMC was graded using the Common Terminology Criteria for Adverse Events (CTCAE) version 5.0 at different times to assess response of IMC to treatment [16]. Onset of IMC was categorized into abrupt (peak IMC grade was recorded at first presentation) or gradual (peak IMC grade was recorded after the first presentation). Improvement of symptoms was defined as significant lessening of clinical symptoms of at least one CTCAE grade, as reported by the patient and treating physician. Rapid improvement was defined as the improvement’s occurring within 72 h of IMC treatment (as recommended by the current guidelines).

### Clinical characteristics

Collected information included patients’ demographics; clinical and oncologic history; and IMC treatment, clinical presentation, and outcomes. Demographics included age at the time of first ICI infusion, gender, and race/ethnicity. Comorbidities according to the Charlson Comorbidities Index were reported [17]. Cancer type, stage, and treatment were documented as well. Type of ICI, number of infusions, duration of treatment, and non-gastrointestinal irAEs were collected. Also, we reported if ICI therapy was resumed after the first IMC episode. Cancer status at the time of staging and before and after SIT treatment was recorded according to Immune-Modified Response Evaluation Criteria In Solid Tumors (imRECIST) and Immune Response Evaluation Criteria In Solid Tumors (iRECIST) [18, 19].

### IMC treatment

Treatment of IMC consisted of steroids and a biologic agent (infliximab or vedolizumab or both). Initial dosage of steroids was given following the recommendations from treatment guidelines for irAEs depending on the grade and responsiveness of IMC [1, 2]. Briefly, 1 mg/kg/day is the recommended initial dose of steroid for grade 2 IMC, and 1–2 mg/kg/day is the recommended initial dose of steroid for grade 3–4 IMC. Steroids were administered orally in grade 2 IMC and intravenously in grade 3–4 IMC. Cumulative duration of steroid treatment was measured, but cumulative dose was not because the dosage varies widely and is impractical to calculate. The number of SIT infusions was measured for both infliximab and vedolizumab. In this study, gradual weaning of steroids was started after the first infusion of SIT. The requirement for hospitalization and intensive care unit admissions due to IMC was documented. Additionally, we reported the number of hospital admissions for IMC and the cumulative duration of associated hospitalizations. Steroid tapering was defined as "failed" if the symptoms recurred after decreasing the dose of steroids from the maximum dose. We also recorded the number of steroid tapering attempts before successful cessation of steroids. Time from IMC onset to SIT initiation was noted as well. Recurrence of IMC after complete discontinuation of immunosuppressive therapy was recorded. Survival duration was defined as the time from ICI initiation to last clinical encounter or death.

### Investigational data

Fecal calprotectin and lactoferrin values before and after immunosuppressive therapy were recorded. Endoscopic and histologic features at the time of IMC presentation were documented. Endoscopic high-risk features of IMC were defined according to our previous study [7]. Briefly, these high-risk features were one or more of the following: ulcers deeper than 2 mm, ulcers wider than 1 cm, and extensive inflammation involving the colon proximal to the splenic flexure [7]. Repeated endoscopic and histologic evaluations to assess for treatment response were reviewed. Endoscopic remission was defined as the healing of the mucosal ulceration or the resolution of mucosal inflammation. Histologic remission was defined as the absence of active inflammatory features on the repeat mucosal biopsies.

### Statistical analyses

Continuous variables were described by mean and standard deviation (SD) or median and interquartile range (IQR). Categorical variables were described by frequencies and percentages. Fisher exact and χ^2^ tests were used to compare categorical variables. The Wilcoxon rank sum test was used to compare continuous variables. Factors associated with IMC recurrence or steroid taper failure were assessed by univariate logistic regression analyses. To estimate and compare survival durations between subgroups, we used Kaplan-Meier curves and log rank tests. All statistical tests were two-sided. *P* values of 0.05 or less were considered statistically significant. Statistical analyses were computed by SAS version 9.4 software and SPSS version 24.0 software.

## Results

### Included patients

During the study period, 1459 patients received ICI; 179 (12%) of them developed IMC of any grade. Of these 179 patients, 84 (47%) received SIT. The mean age of these 84 patients was 60 years (SD, 14 years). The predominant gender was male (*n* = 66 [79%]), and the predominant ethnicity was Caucasian (*n* = 76 [91%]). Melanoma was the most common malignancy (*n* = 40 [48%]), followed by genitourinary cancer (*n* = 28 [33%]) and thoracic, head, and neck cancers (11 [13%]). Most patients had stage IV malignancy (*n* = 72 [87%]). ICI therapy consisted of inhibitors of programmed death protein-1 or its ligand in 33 patients (39%), inhibitors of cytotoxic T-lymphocyte associated protein-4 in 21 patients (25%), and a combination of ICIs in 30 patients (36%). The median clinical follow-up duration of this study was 5 months (range, 1–10 months), and the median endoscopic follow-up time was 4 months (range, 1–7 months).

### IMC features at time of SIT initiation

**Fig. 1:**
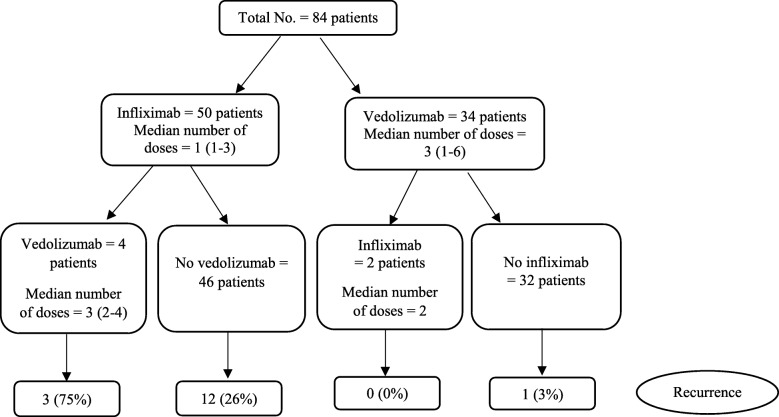
Patient characteristics by SIT

According to the peak grade of diarrhea, 57 (68%) patients had grade 3 and 13 (16%) had grade 4. Regarding colitis grade, 55% of patients had grade 2 and 45% of patients had grade 3–4. Laboratory assessment of fecal lactoferrin was performed in 56 patients, 53 (95%) of them had positive test. Fecal calprotectin value was obtained in 40 patients with a median of 235 (IQR, 91–479). Of the 64 patients who had endoscopic evaluation with biopsy, 40 (63%) had high-risk features of IMC on endoscopy and 60 (94%) had features of active inflammation on histology. SIT treatment consisted of infliximab in 50 patients (60%) and vedolizumab in 34 patients (40%, Fig. 1).

### Timing of SIT initiation

The median time from IMC onset to SIT initiation was 10 days (IQR, 5–23 days). Patients who received SIT within 10 days of IMC onset (*n* = 44 [52%]) required fewer hospitalizations (*P* = 0.026), had shorter duration of symptoms (*P* = 0.002), were less likely to experience steroid taper failure after SIT therapy (*P* = 0.033), and consequently had fewer failed steroid tapering attempts (*P* < 0.001) compared with patients who received SIT after 10 days of IMC onset (40 [48%]; Table 1). The two groups had similar severity of diarrhea and colitis, endoscopic and histologic features, and ICI type.

**Table 1:** Clinical characteristics stratified by the timing of selective immunosuppressive therapy initiation (SIT)

Covariate	≤ 10 days of onset *N* = 44	> 10 days of onset *N* = 40	*P*
ICI type, No. (%)			0.687
Anti-CTLA-4 monotherapy	11 (25)	10 (25)	
Anti-PD-1/L1 monotherapy	19 (43)	14 (35)	
Combination	14 (32)	16 (40)	
Diarrhea grade, No. (%)			0.668
1–2	8 (18)	6 (15)	
3	28 (64)	29 (73)	
4	8 (18)	5 (13)	
Colitis grade, No. (%)			0.603
1–2	24 (56)	22 (55)	
3	16 (37)	17 (43)	
4	3 (7)	1 (3)	
Endoscopic features, No. (%)			0.739
Ulcer	13 (42)	17 (52)	
Non-ulcerative inflammation	12 (39)	11 (33)	
Normal	6 (19)	5 (15)	
High-risk endoscopic features initially, No. (%)^a^	17 (55)	23 (70)	0.302
Overall duration of steroids, mean days (SD)	64 (38)	82 (51)	0.092
Duration of hospitalization, mean days (SD)	10 (8)	12 (8)	0.321
Duration of symptoms, mean days (SD)	25 (32)	50 (40)	0.002
Follow-up duration, mean months (SD)	5 (3)	4 (3)	0.875
Number of steroids tapering attempts, median (IQR)	1 (1–4)	2 (1–4)	< 0.001
Multiple hospitalization, No. (%)	13 (30)	22 (55)	0.026
Failed steroid tapering after SIT, No. (%)^b^	9 (23)	19 (49)	0.033
Recurrent IMC, No. (%)	8 (18)	8 (20)	1.000
Infectious adverse events, No. (%)	16 (36)	9 (23)	0.233

^a^High-risk features are ulcers deeper than 2 mm or wider than 1 cm, and extensive endoscopic inflammation involving the colon proximal to the splenic flexure

^b^Available for the 79 patients who received steroids

Abbreviation: SIT, selective immunosuppressive therapy

### Steroid tapering

Steroids were weaned successfully without return of IMC symptoms after SIT therapy in 50 (60%) patients. Compared with these patients, patients in whom steroid tapering failed after SIT had higher peak grades of IMC (*P* = 0.016), were more likely to have a positive fecal lactoferrin test at time of IMC onset (*P* = 0.028), were more likely to present with mucosal ulceration (*P* = 0.015), had longer duration of IMC symptoms (*P* < 0.001), received longer duration of steroid treatment (*P* < 0.001), had slower improvement in response to steroid treatment before SIT (*P* = 0.009), were more likely to experience failed steroid therapy before SIT (*P* = 0.002), initiated SIT later (*P* = 0.002), were hospitalized for longer durations (*P* = 0.001), and were more likely to require multiple hospitalizations (*P* < 0.001; Table 2).

**Table 2:** Clinical characteristics stratified by the resistance to steroid

Covariate	Failed steroid tapering after SIT *N* = 28	Successful steroid tapering after SIT *N* = 50	*P*
Diarrhea grade, No. (%)			0.087
1–2	1 (4)	11 (22)	
3	21 (75)	32 (64)	
4	6 (21)	7 (14)	
Colitis grade, No. (%)			0.016
1–2	9 (32)	33 (66)	
3	17 (61)	15 (30)	
4	2 (7)	2 (4)	
Symptom onset, No. (%)			0.478
Abrupt	11 (39)	25 (50)	
Gradual	17 (61)	25 (50)	
Positive lactoferrin at time of onset, No. (%)	22 (79)	26 (52)	0.028
Overall duration of steroids, mean days (SD)	99 (46)	58 (39)	< 0.001
Duration of hospitalization, mean days (SD)	15 (9)	9 (6)	0.001
Duration of symptoms, mean days (SD)	72 (42)	19 (19)	< 0.001
Endoscopic features, No. (%)			0.015
Ulcer	15 (60)	12 (35)	
Non-ulcerative inflammation	4 (16)	18 (53)	
Normal	6 (24)	4 (12)	
Duration from onset to SIT, mean days (SD)	28 (32)	12 (11)	0.002
Calprotectin at time of onset, mean (SD)	363 (329)	311 (272)	0.610
Rapid improvement with steroid before SIT, No. (%)	8 (29)	31 (62)	0.009
Failed steroid tapering before SIT, No. (%)^a^	23 (82)	22 (44)	0.002
Multiple hospitalizations, No. (%)	21 (75)	13 (26)	< 0.001
Recurrent IMC, No. (%)	10 (36)	6 (12)	0.019

^a^Available for the 79 patients that received steroids

Abbreviation: SIT, selective immunosuppressive therapy

### Number of SIT infusions

Most patients received one or two infusions of SIT (*n* = 54 [64%]); the median number of infusions was 3 (IQR, 3–4). Compared with patients who received three or more SIT infusions, patients who received one or two SIT infusions were more likely to experience failure of steroid tapering and recurrent IMC (*P* = 0.030 and *P* = 0.008, respectively; Table 3). Additionally, patients who received one to two SIT infusions commonly received infliximab rather than vedolizumab (*P* < 0.001) and had higher levels of fecal calprotectin after SIT (*P* = 0.011). However, the peak CTCAE grades of diarrhea and colitis were comparable between the two groups.

**Table 3:** IMC outcomes stratified by the number of SIT doses

Covariate	1–2 doses *N* = 54	≥ 3 doses *N* = 30	*P*
Multiple hospitalization, No. (%)	25 (46)	10 (33)	0.356
Failed steroid tapering after SIT, No. (%)^a^	13 (27)	15 (52)	0.030
Infliximab, No. (%)	49 (91)	3 (10)	< 0.001
Endoscopic features, No. (%)			0.388
Ulcer	17 (49)	13 (45)	
Non-ulcerative inflammation	14 (40)	9 (31)	
Normal	4 (11)	7 (24)	
Endoscopic remission, No. (%)^b^	9 (64)	14 (67)	1.000
Histologic remission, No. (%)^b^	7 (44)	17 (71)	0.087
Infectious adverse events, No. (%)	16 (30)	9 (30)	1.000
Recurrent IMC, No. (%)	15 (28)	1 (3)	0.008
Calprotectin after SIT, mean (SD)	312 (325)	118 (138)	0.011
Mean calprotectin difference before and after SIT, (SD)	234 (204)	222 (165)	0.882

^a^Available for the 79 patients who received steroids

^b^Available for 40 patients

Abbreviation: SIT, selective immunosuppressive therapy

### Response to steroid treatment

Of the 79 patients who received steroids before SIT, 38 had improvement of IMC symptoms and 41 had no improvement. Compared to patients who had improvement of IMC symptoms with corticosteroid treatment before SIT, patients whose symptoms did not improve on steroids received steroids for a longer duration (*P* < 0.001), were more likely to experience steroid taper failure after SIT (*P* = 0.010), and consequently required more steroid tapering attempts (*P* = 0.014) and were more likely to be hospitalized multiple times (*P* = 0.051) and for longer durations (*P* = 0.001, Table 4). This difference in clinical outcomes was seen despite the fact that the two groups had similar grades of diarrhea and colitis.

**Table 4:** Response to steroid treatment before SIT and outcomes

Covariate	Improvement with steroid before SIT *N* = 38	No improvement with steroid before SIT *N* = 41	*P*
Multiple hospitalization, No. (%)	12 (38)	23 (64)	0.051
Failed steroid tapering after SIT, No. (%)	8 (21)	20 (50)	0.010
Overall duration of steroids, mean days (SD)	63 (40)	113 (45)	< 0.001
Duration of hospitalization, mean days (SD)	9 (6)	17 (11)	0.001
Duration of symptoms, mean days (SD)	25 (26)	83 (42)	< 0.001
Number of steroids tapering attempts, No. (%)			0.014
1	24 (63)	11 (28)	
2–4	14 (37)	29 (73)	
Endoscopic remission, No. (%)	10 (67)	13 (65)	1.000
Histologic remission, No. (%)^a^	11 (73)	13 (52)	0.318
Recurrent IMC, No. (%)	5 (13)	11 (27)	0.166

^a^Available for 40 patients

Abbreviation: SIT, selective immunosuppressive therapy

Among the 41 patients who had poor response to steroids before SIT, 17 began SIT within 10 days and 24 after 10 days of IMC onset. In these patients, early introduction of SIT was associated with a lower number of hospitalizations (*P* = 0.014; Table 5). Among the 38 patients who had good response to steroids before SIT, 23 received SIT within 10 days and 15 more than 10 days after IMC onset. In these patients, early introduction of SIT was associated with less resistance to steroid tapering after SIT (*P* = 0.003), fewer failed steroid tapering attempts (*P* < 0.001), and shorter duration of symptoms (*P* = 0.016).

**Table 5:** Effect of timing of SIT initiation after IMC onset on outcomes among patients who had different response to steroid therapy

Covariate	Poor response to steroid			Good response to steroid		
	≤ 10 days *N* = 17	> 10 days *N* = 24	*P*	≤ 10 days *N* = 23	> 10 days *N* = 15	*P*
Multiple hospitalization, No. (%)	7 (41)	16 (84)	0.014	6 (32)	6 (46)	0.473
Failed steroid tapering after SIT, No. (%)	8 (50)	12 (50)	1.000	1 (4)	7 (47)	0.003
Recurrent IMC, No. (%)	4 (24)	7 (29)	0.736	4 (17)	1 (7)	0.630
Number of steroids tapering attempts, No. (%)			0.532			< 0.001
1	6 (38)	5 (21)		20 (87)	4 (27)	
2–4	10 (62)	19 (79)		3 (13)	11 (73)	
Overall duration of steroids, mean days (SD)	73 (43)	96 (57)	0.184	58 (34)	59 (30)	0.942
Duration of hospitalization, mean days (SD)	12 (9)	13 (8)	0.545	9 (6)	10 (8)	0.571
Duration of symptoms, mean days (SD)	39 (38)	57 (45)	0.179	17 (24)	39 (30)	0.016

Abbreviation: SIT, selective immunosuppressive therapy

### Duration of steroid treatment

In our cohort, 16 patients received steroids for less than 6 weeks and began SIT within 10 days of IMC onset. These patients, compared with the 30 patients who received steroids for longer than 6 weeks and began SIT more than 10 days after IMC onset, had shorter duration of symptoms (*P* < 0.001), had fewer steroid tapering attempts (*P* < 0.001), and were hospitalized less frequently (*P* < 0.001) and for shorter duration (*P* = 0.034; Additional file 1: Table S1).

### IMC recurrence

Sixteen patients (19%) developed IMC recurrence after SIT therapy and complete steroid weaning. Logistic regression analysis showed that the predictors of IMC recurrence included failure of steroid weaning after SIT (*P* = 0.017), infliximab therapy rather than vedolizumab therapy (*P* = 0.017), 2 or fewer infusions of SIT compared with 3 or more infusions (*P* = 0.023), multiple hospitalizations (*P* = 0.002), persistent endoscopic (*P* = 0.025) and histologic (*P* = 0.033) inflammation after SIT, lower number of steroid tapering attempts (*P* = 0.001), shorter duration of steroids (*P* = 0.022), higher calprotectin values after SIT (*P* = 0.014), longer duration of symptoms (*P* = 0.008), and longer duration of hospitalization (*P* = 0.001; Table 6).

**Table 6:** Outcomes after SIT

Characteristics	Failed steroid tapering after SIT		IMC recurrence	
	OR (95% CI)	*P*	OR (95% CI)	*P*
ICI type				
Anti-PD-1/L1 monotherapy	Reference		Reference	
Anti-CTLA-4 therapy	0.50 (0.19–1.29)	0.150	1.09 (0.36–3.37)	0.871
Colitis grade				
1–2	Reference		Reference	
3–4	4.09 (1.53–10.98)	0.005	1.79 (0.59–3.38)	0.299
Multiple hospitalizations	9.05 (2.79–29.33)	< 0.001	26.25 (3.22–213.82)	0.002
Failed steroid tapering before SIT	3.75 (1.39–10.16)	0.009	2.42 (0.75–7.77)	0.138
Failed steroid tapering after SIT	–	–	4.07 (1.29–12.88)	0.017
Type of SIT				
Vedolizumab	Reference		Reference	
Infliximab	0.47 (0.18–1.22)	0.120	12.57 (1.57–100.57)	0.017
No. of SIT infusions				
1–2	Reference		Reference	
≥3	2.97 (1.13–7.79)	0.027	0.09 (0.01–0.72)	0.023
Endoscopic remission	1.68 (0.41–6.96)	0.474	0.15 (0.03–0.79)	0.025
Histologic remission	0.67 (0.18–2.46)	0.543	0.18 (0.04–0.88)	0.033
Number of steroids tapering attempts	9.50 (3.76–24.01)	< 0.001	3.35 (1.68–6.69)	0.001
Duration from IMC onset to SIT	1.05 (1.01–1.09)	0.011	1.00 (0.98–1.03)	0.774
Overall duration of steroids	1.02 (1.01–1.04)	0.001	1.01 (1.00–1.03)	0.022
Calprotectin at time of onset	1.00 (0.99–1.00)	0.599	1.00 (0.99–1.01)	0.346
Calprotectin after SIT	1.00 (0.99–1.00)	0.573	1.01 (1.00–1.01)	0.014
Duration of hospitalization	1.12 (1.04–1.21)	0.004	1.14 (1.05–1.23)	0.001
Duration of symptoms	1.06 (1.03–1.08)	< 0.001	1.02 (1.01–1.03)	0.008

Abbreviations: CI, confidence interval; CTLA, cytotoxic T lymphocyte associated protein; SIT, selective immunosuppressive therapy**;** OR, odds ratio; PD-1, programmed death protein 1; PD-L1, PD-1 ligand

### ICI resumption and IMC recurrence

Fourteen patients resumed ICI after IMC resolution. Eight of them received vedolizumab concurrently with ICI infusions and six did not (Additional file 1 Figure S1). Only one patient who received concurrent vedolizumab experienced IMC recurrence, whereas three patients who did not receive vedolizumab experienced IMC recurrence. IMC recurrence necessitated immunosuppressive therapy in the one patient who received vedolizumab and in two of the patients who did not.

### Survival analyses

At the end of study period, 12 patients were dead; 11 because of cancer progression and one because of hypophysitis and sepsis. Survival analyses indicated a comparable impact of vedolizumab and infliximab on overall survival rates (*P* = 0.151; Additional file 1 Figure S2). Patients who had their ICI treatment course interrupted early (i.e., one or two infusions) had worse survival rates compared with patients who received ICI for more than three or more infusions (*P* = 0.008; Additional file 1 Figure S3).

## Discussion

In the era of immunotherapy, precise and evidence-based treatment recommendations for adverse events related to ICIs become critical to attaining the greatest benefit of their use, since IMC could sometimes be correlated to a clinical benefit from ICI therapy [20–22]. Current treatment guidelines recommend SIT only in patients with steroid-refractory IMC, with no recommendation regarding the number of SIT infusions [1–4]. In an effort to improve on the current IMC management guidelines, and in light of analogy with the treatment of IBD where SIT could be introduced as frontline treatment, almost half our patients were treated according to a tailored treatment algorithm devised at our institution [23] and derived from IBD management strategies instead of the current IMC management recommendations. This algorithm recommends early introduction of SIT regardless of steroid responsiveness. Furthermore, three or more infusions of SIT are recommended. The rest of our cohort received IMC treatment according to the published guidelines [1, 2]. This approach allowed us to compare the outcomes of both groups to assess the applicability of IBD treatment to patients with IMC.

In this study, the decision to initiate SIT was made based on clinical, endoscopic and histologic features, including unresponsiveness of IMC symptoms to steroids, high grade of IMC, large deep mucosal ulceration on endoscopy, and active histologic inflammation [7]. Of note, most of these features coexisted in patients who received SIT at time of IMC onset.

We investigated the utility of early SIT in the treatment of IMC, a strategy adopted from the management modalities studied and validated in patients with IBD [24–27]. In this study, we found that the introduction of SIT early during the course of IMC, i.e., without waiting for response to corticosteroids, was associated with a favorable impact on the disease course, response of IMC to therapy, reduced length of IMC-related hospital stay, and reduced need for re-hospitalization. Despite complete resolution of symptoms in most patients after one infusion of infliximab or vedolizumab, three or more infusions of SIT led to a lower IMC recurrence rate compared with one or two infusions.

These findings highlight the similarities between IMC and IBD. 1) There is a potential discrepancy between clinical response and true endoscopic remission in IMC; a similar distinction is seen in IBD. This discrepancy challenges the current definition of disease remission in IMC. 2) Early introduction of SIT leads to improved response and outcomes in both IMC and IBD. 3) There is a role for a dose regimen that first induces IMC remission (the first one to two doses of SIT) and then maintains remission with subsequent dosing. We speculate that a maintenance regimen of SIT over several weeks, as is done in IBD, helps achieve mucosal healing (beyond the initial clinical response) and allows the effects of immunotherapy on the gut to dissipate over time. This could explain the positive outcomes seen in IMC patients who received SIT early and for more than two infusions, in terms of fewer hospitalizations, fewer instances of IMC recurrence, and a higher overall survival rate.

Our current study found the introduction of SIT within 10 days of IMC onset to be associated with shorter duration of symptoms, less requirement for hospitalization, and lower dependence on steroids after SIT compared with late introduction of SIT. To account for the potential confounding effect that corticosteroid therapy responsiveness could have introduced, we compared patients with steroid-responsive and steroid-refractory IMC separately, followed by stratification of patients who received SIT within 10 days of IMC and those who did not. Our findings persisted, even though the results for steroid responders were more robust. Another analysis showed that receiving three or more doses of SIT was associated with a lower recurrence rate, lower calprotectin levels at the end of SIT treatment, fewer patients who experienced steroid taper failure, and more patients who experienced histologic remission. These findings validate the efficacy of our treatment algorithm in decreasing the disease burden on patients and improving response to therapy.

Though symptomatic resolution is a common clinical endpoint in treating patients with colitis, it may not necessarily translate into actual disease remission. It has been shown in the setting of IBD that symptoms do not correlate with disease activity, and there is preliminary evidence to suggest the same in the setting of IMC. In this study, we observed a decrease in the risk of IMC recurrence in patients who achieved endoscopic and histologic remission after SIT in addition to clinical symptomatic resolution. Therefore, follow-up colonoscopy after SIT to evaluate for endoscopic remission before stopping therapy is crucial to decrease the risk of IMC recurrence.

The fecal microbiome has been proposed to play a role in the activity of ICI and the development of IMC [28–31]. We previously performed fecal microbiota transplantation as a novel treatment modality for IMC that is refractory to SIT [32]. This approach achieved great success in cases that were unresponsive to SIT, with quick clinical remission and almost complete resolution of endoscopic and histologic inflammation. Additionally, predictors of unresponsive IMC were found to be a lack of response to steroid therapy within 72 h of IMC onset, endoscopic mucosal ulcerations, high fecal calprotectin values, positive lactoferrin, and high grades of IMC. We therefore recommend the use of fecal microbiota transplantation in patients who are forecasted to have challenging IMC, especially those who have more than one of the aforementioned predictors. However, further validation of our findings is needed.

Steroids are associated with significant morbidity, particularly if used for a prolonged time and given in high doses [33, 34]. The treatment guidelines for IMC recommend at least 6 weeks of steroids. We compared patients who received steroids for more than 6 weeks and SIT more than 10 days after IMC onset (per current IMC guidelines) with those who received steroids for less than 6 weeks and SIT within 10 days of IMC (adopted from our institutional algorithm). Our algorithm was associated with a statistically significant improvement in disease course, response to therapy, length of hospital stay, and frequency of re-hospitalization. Nonetheless, studies that investigate the appropriate length of steroid treatment are warranted.

The primary aim of our group is to provide appropriate control of IMC to sustain ICI therapy, and consequently hinder any negative effect on cancer outcomes. Our survival analysis revealed that patients who received more than three infusions of ICI had better overall survival rates compared with patients who had fewer than three infusions. This emphasizes the importance of sustained ICI therapy.

In our cohort, a few patients resumed ICI therapy after IMC resolution. Vedolizumab was given to a subset of them as a concurrent treatment to prevent recurrence of IMC. Most patients treated with this combination approach did not experience IMC recurrence. Nevertheless, the small number of patients limited our ability to draw any solid conclusions from this result. Further prospective investigation of the utility of SIT in the prevention of IMC recurrence after ICI resumption is hence warranted. A notable finding in our study is the increased recurrence rate with infliximab infusion. Nonetheless, this is probably a result of inadequate number of infusions given to patients that received infliximab rather than low efficacy of infliximab.

Our study has several limitations. The retrospective study design may provide suboptimal details of patients’ medication regimens. Second, the nonrandomized approach limits the strength of our findings. Third, the small sample size limits our ability to perform subgroup analyses with adequate power. Finally, our cohort consisted of patients with various cancer types that were treated by different ICI classes. This heterogeneity indicates that we should not assume that IMC caused by all ICIs behave in a similar fashion.

## Conclusion

Our data suggest that SIT should be initiated early after diagnosis of IMC and not held until failure of steroid therapy or steroid taper. SIT should be given for at least three doses to improve clinical outcomes of IMC and prevent IMC recurrence. Endoscopic and histological remission serves as a better treatment target for IMC compared with clinical remission to ensure a lower incidence of recurrence. These findings need to be validated by large-scale prospective studies to investigate the appropriate duration of steroid therapy and timing of SIT initiation.

## Additional file

Additional file 1: **Table S1.** Effect of steroid treatment duration and timing to SIT initiation after IMC onset. **Figure S1.** Recurrence of IMC after ICI resumption. **Figure S2.** Kaplan-Meier curve showing overall survival duration of patients who received vedolizumab only compared with that of patients who received infliximab with or without vedolizumab. **Figure S3.** Kaplan-Meier curve showing overall survival duration of patients who developed IMC after more than three infusions of ICI compared with those who developed IMC after three or fewer infusions. (DOCX 61 kb)

## Abbreviations

CTCAE: Common Terminology Criteria for Adverse Events
IBD: Inflammatory bowel disease
ICI: Immune checkpoint inhibitor
IMC: Immune-mediated colitis
imRECIST: immune-Modified Response Evaluation Criteria In Solid Tumors
irAE: immunotherapy-related adverse event
iRECIST: immune-related Response Evaluation Criteria in Solid Tumors
SIT: Selective immunosuppressive therapy
