# Supplementary material for: Early introduction of selective immunosuppressive therapy associated with favorable clinical outcomes in patients with immune checkpoint inhibitor–induced colitis
Source: J Immunother Cancer. 2019 Apr 2;7:93. doi: 10.1186/s40425-019-0577-1 (PMC6444537; doi:10.1186/s40425-019-0577-1)
Supplement: Supplementary file 1 — Table S1. Effect of steroid treatment duration and timing to SIT initiation after IMC onset. Figure S1. Recurrence of IMC after ICI resumption. Figure S2. Kaplan-Meier curve showing overall survival duration of patients who received vedolizumab only compared with that of patients who received infliximab with or without vedolizumab. Figure S3. Kaplan-Meier curve showing overall survival duration of patients who developed IMC after more than three infusions of ICI compared with those who developed IMC after three or fewer infusions. (DOCX 61 kb) [file 40425_2019_577_MOESM1_ESM.docx]

**Table S1.** Effect of steroid treatment duration and timing to SIT initiation after IMC onset.

| **Covariate** | **Steroid ≤ 42 days and SIT ≤ 10 days**  **N = 16** | **Steroid > 42 days and SIT > 10 days**  **N = 30** | **P value** |
| --- | --- | --- | --- |
| Multiple hospitalizations, No. (%) | 1 (7) | 19 (73) | < 0.001 |
| Rapid improvement with steroid before SIT, No. (%) | 10 (63) | 10 (33) | 0.070 |
| Duration of hospitalization, mean days, (SD) | 8 (5) | 13 (8) | 0.034 |
| Duration of symptoms, mean days (SD) | 8 (6) | 60 (41) | < 0.001 |
| Failed steroid tapering after SIT, No. (%)^a^ | 0 (0) | 18 (60) | < 0.001 |
| Number of steroids tapering attempts, median, No. (%) |  |  | < 0.001 |
| 1 | 16 (100) | 4 (13) |  |
| 2-4 | 0 (0) | 26 (87) |  |

^a^Available for the 78 patients that received steroids.

^b^Available for 40 patients.

Abbreviation: SIT, selective immunosuppressive therapy.

**Figure S1.** ICI resumption.

PD-1/L1 resumption

= 14

1 (100%)

2 (67%)

3 (50%)

No concurrent vedolizumab = 6

Concurrent vedolizumab = 8

1 (13%)

**Figure S2**. Overall survival curve by the type of selective immunosuppressive therapy.

**Figure S3.** Overall survival curve by the time of ICI therapy interruption.
